# The relationship between adolescent sleep duration and exposure to school bullying: the masking effect of depressive symptoms

**DOI:** 10.3389/fpsyg.2024.1417960

**Published:** 2024-12-16

**Authors:** Rushuang Zeng, Dang Han, Wei Du, Jing Wen, Youxian Zhang, Zongyu Li, Qun Du, Yan Qi, Yu Li, Jia He

**Affiliations:** ^1^Key Laboratory of Xinjiang Endemic and Ethnic Diseases of the Ministry of Education, Department of Public Health, Shihezi University School of Medicine, Xinjiang, China; ^2^Key Laboratory for Prevention and Control of Emerging Infectious Diseases and Public Health Security, The Xinjiang Production and Construction Corps, Xinjiang, China; ^3^West China Hospital of Sichuan University, Chengdu, Sichuan, China; ^4^The Center for Disease Control and Prevention in the 2nd Division of Xinjiang Production and Construction Corps, Xinjiang, China; ^5^Public Health Management Center, Yanqi Hospital in the 2nd Division of Xinjiang Production and Construction Corps, Xinjiang, China

**Keywords:** depressive symptoms, sleep duration, masking effect, adolescents, school bullying

## Abstract

**Background:**

Adolescents who suffer from school bullying are often accompanied by problems such as sleep disorders and depression. However, it remains unclear how depressive symptoms and sleep assessments such as sleep duration interact in the specific mechanisms of exposure to school bullying.

**Objective:**

To understand the role of sleep duration, depressive symptoms on school bullying in adolescents and the mediating role of sleep duration in this context.

**Methods:**

A total of 1730 adolescents were selected from Xinjiang province, China by stratified cluster random sampling in 2020, and their demographic characteristics, exposure to school bullying, depressive symptoms, and sleep duration were investigated by questionnaire. Multifactorial logistics regression analyses were performed to examine the effects of sleep duration and depressive symptoms on school bullying. Furthermore, the bootstrap method was used to explore the mediating effect and masking effect of depressive symptoms between sleep duration and school bullying by PROCESS macro in SPSS 26.0.

**Results:**

A total of 16.42% of adolescents suffered from school bullying and 12.25% showed depressive symptoms. Multifactorial logistics regression analyses revealed that possible depression and depression increase the risk of exposure to school bullying compared to normal group. Sleep duration less than 8 h and between 8 and 10 h are protective factors for exposure to three types of school bullying relative to those who sleep more than 10 h. Additionally, sleep duration affected exposure to school bullying through depressive symptoms (*β* = 0.011) and depressive symptoms masked the effect between sleep duration and exposure to school bullying with an effect of 60.17%. The masking effect remained stable after adjusting for gender, age, ethnicity, body mass index, and exercise intensity (indirect effect = −0.017, 95%CI: −0.026 to −0.009).

**Conclusion:**

Depressive symptoms may be a risk factor for exposure to school bullying, while moderate and inadequate sleep duration may be protective factors for exposure to all three types of school bullying simultaneously. The effect of sleep duration on adolescents’ exposure to school bullying was affected by the masking effect of depressive symptoms, and appropriate sleep duration and a positive and healthy mindset were beneficial in reducing the occurrence of school bullying.

## 1 Introduction

School bullying is a significant public health problem increasing international attention (Xin et al., 2024), and the reported rate of school bullying in China ranges from 2 to 66% (Xu et al., 2022). Considering its significant impact on adolescents’ physical and mental health, addressing school bullying demands focused efforts from educators and government agencies (Penghui and Mengfan, 2024).

Insufficient sleep is common among adolescents, often attributed to heavy academic demands (Liang et al., 2021). Sleep duration is critical to sleep quality and is closely tied to neurocognitive development and mental health (Owens, 2014; Speyer, 2022). Studies have shown that adolescents exposed to school bullying report more sleep problems compared to their peers (Ding et al., 2023; Hasan et al., 2021), although a previous study found no significant correlation between bullying exposure and sleep duration (Ranum et al., 2021). This inconsistency highlights the need for further exploration. Substantial evidence suggests that school bullying places adolescents at higher risk for mental health issues such as depression and anxiety, with severe cases leading to self-harm or suicide (Xu et al., 2022; Wang et al., 2015; Guo et al., 2020; Martínez-Monteagudo et al., 2020). Conversely, depressive symptoms are also linked to increased vulnerability to school bullying (Li et al., 2023). Gireesh et al. highlighted that depressive symptoms reduce adolescents’ wellbeing, making them more susceptible to victimization (Gireesh et al., 2018). Thus, depressive symptoms may act as both a risk factor for and a consequence of school bullying.

Additionally, sleep duration has been shown to mediate the relationship between bullying victimization and depressive symptoms (Kwon et al., 2020; Mei et al., 2021). However, the exact relationship between sleep duration and school bullying exposure remains unclear. A previous study demonstrated that excessive exposure to bullying may place adolescents at higher risk for psychological distress and thus lead to shorter sleep duration (Sampasa-Kanyinga et al., 2018). While these findings emphasize the heightened risk of sleep and psychological issues among bullied adolescents, the role of depressive symptoms as a potential mediator between sleep duration and bullying exposure remains underexplored.

Mediating effect models have been widely used to examine how mental health and social factors influence school bullying exposure (Xiao et al., 2022; Huang et al., 2024). Building on this framework, our study aims to explore whether depressive symptoms mediate the relationship between sleep duration and school bullying exposure. By constructing a mediation model, we seek to provide evidence-based insights to inform prevention and intervention strategies for school bullying.

## 2 Methods

### 2.1 Participants and procedure

Stratified cluster random sampling was employed to select two urban and two rural schools in Xinjiang province, China in this cross-sectional study, encompassing both middle and high schools between August and October 2020. Inclusion criteria for this study were: (1) students enrolled in middle or high schools in urban or rural areas of Tiemenguan, Xinjiang province; (2) students who were willing to participate and whose guardians provided informed consent; (3) students who had not participated in any other similar study on bullying or mental health during the current academic year. Exclusion criteria were: (1) students absent on the day of data collection; (2) students with severe physical or mental conditions that would prevent them from completing the survey; (3) students who declined participation or whose guardians did not provide consent. A minimum of 80 students from each grade were randomly selected to complete the questionnaire, with any shortfalls being addressed by drawing additional participants from nearby schools of the same type. The selected sample of middle and high school students is considered representative of the broader adolescent population in Xinjiang, as these groups are the primary demographic affected by school bullying. To ensure the sample’s representativeness, we compared the sociodemographic characteristics of the respondents with regional census data from Xinjiang. Out of the 1,971 students initially sampled, 1,730 completed the survey, yielding a response rate of 87.77%. Informed consent was obtained from all participants and their guardians, and they willingly participated in the survey.

### 2.2 Physical examination and questionnaire survey

Physical examinations were conducted by trained personnel using standardized measurement instruments. Body mass index (BMI) was calculated as weight (kg) divided by height squared (m^2^), and categorized as follows: BMI < 18.5 kg/m^2^ was classified as underweight, 18.5 ≤ BMI < 24.0 kg/m^2^ as normal weight, and BMI ≥ 24.0 kg/m^2^ as overweight (2022). The questionnaires included sections on demographic information, medical history, sleep duration, bullying victimization, and self-assessment scales for depressive symptoms.

#### 2.2.1 Sleep duration

Adolescents included in this study used their self-reported sleep duration as a variable in this study. According to the prescribed standards of appropriate sleep duration for adolescents, adolescents are categorized into three groups: less than 8 h, between 8 and 10 h, and more than 10 h (Chen et al., 2014).

#### 2.2.2 Bullying victim questionnaire

The Olweus Bully-Victim Questionnaire was used in the survey, which is widely employed among Chinese adolescents (Thomas et al., 2017). Each question had three response options (never, sometimes, often), and respondents who answered “sometimes” or “often” to one or more questions were classified as having experienced bullying in school. The questionnaire assesses bullying-related behaviors occurring within the past 30 days, including three forms of physical bullying, verbal bullying, and emotional neglect, with a total of six questions (Qian et al., 2022). The Cronbach’s *α* coefficient for this scale was 0.795, indicating good internal consistency reliability. In addition, value of Kaiser-Meyer-Olkin (KMO) was 0.819 (*p* < 0.001), reflecting good structural validity.

#### 2.2.3 Depressive symptoms

The Center for Epidemiologic Studies Depression Scale (CES-D), developed by the National Institute of Psychiatry, was used to assess depressive symptoms in adolescents (Cosco et al., 2020; Roth et al., 2008). The scale yields scores ranging from 0 to 60, with a total score of 16–19 indicating “possible depression” and scores greater than 20 classified as “depression” (Vilagut et al., 2016), and a score of 16 or higher is indicative of “depressive symptoms” (Andresen et al., 1994). The reliability and validity of each item in the CES-D were excellent with (Cronbach’s α: 0.801; value of KMO: 0.908, *p* < 0.001).

### 2.3 Quality control

For each school and field survey team, 5% of the daily workload was randomly selected for retesting to ensure the integrity and accuracy of the database. Upon completion of the survey, we conducted a consistency check on the double-entry database submitted by each school. If the compliance rate of the consistency test fell below 90%, all data were re-entered.

### 2.4 Statistical analysis

Basic demographic characteristics were compared between groups using descriptive statistical analysis, and multifactorial logistic regression analyses were conducted to evaluate the influence of sleep duration and depressive symptoms on school bullying. The Harman one-factor test was applied to assess the reliability of study items. Additionally, Model 4 in the PROCESSv3.3 macro developed by Hayes was utilized to examine mediating effects. A masking effect was identified when both the total and indirect effects were significant and the absolute value of the direct effect exceeded that of the total effect, and a discrepancy was observed between the indirect and direct effects. Standard errors and confidence intervals for parameter estimates were calculated using the Bootstrap method with 5,000 resamples. Results with 95% confidence intervals excluding 0 and *p* < 0.05 were deemed statistically significant. All data were double-entered using EpiData 3.1 for quality assurance, and statistical analyses were performed in SPSS 26.0.

## 3 Results

### 3.1 Baseline characteristics of exposure to school bullying

Among 1,730 adolescents, the prevalence of school bullying was 16.42%, with higher rates observed in boys (20.06%), junior high school students (18.57%), rural students (22.63%), and non-resident students (18.90%) (*p* < 0.01). The prevalence of school bullying increased significantly with the severity of depressive symptoms (χ^2^ trend = 73.196, *p* < 0.01). Adolescents with depressive symptoms exhibited the highest rate of exposure to school bullying (33.96%) (Table 1).

**Table 1:** Comparison of exposure to school bullying reporting rates for different groups of students.

Variables	Group	Number	With bullying (x ± s/%)	Without bullying (x ± s/%)	t/χ^2^	*p*
Sleep duration (h)		1730	7.642 ± 1.196	7.550 ± 1.085	1.289	0.198
Total score of CES-D		1730	15.725 ± 9.865	9.909 ± 7.382	9.432	< 0.001
Gender	Male	907	182 (20.06)	725 (79.93)	18.511	< 0.001
	Female	823	102 (12.39)	721 (87.61)		
Grade	Junior high	980	182 (18.57)	798 (81.42)	7.653	0.006
	Senior high	750	102 (13.60)	648 (86.40)		
Ethnicity	Han Chinese	1,623	264 (16.27)	1,359 (83.73)	0.430	0.512
	Other	107	20 (18.69)	87 (81.31)		
Monitoring point	Urban area	1,063	133 (12.51)	930 (87.49)	30.632	< 0.001
	Rural area	667	151 (22.63)	516 (77.36)		
Living in the school	Yes	857	119 (13.88)	738 (86.11)	7.926	0.005
	No	873	165 (18.90)	708 (81.10)		
Exercise intensity	0 days	253	40 (15.81)	213 (84.18)	1.055	0.590
	1–3 days	709	124 (17.49)	585 (82.51)		
	4–7 days	768	120 (15.63)	648 (84.37)		
BMI	Thin	471	75 (15.92)	396 (84.08)	2.063	0.356
	Normal	991	157 (15.84)	834 (84.15)		
	Overweight	268	52 (19.40)	216 (80.60)		
Depressive symptoms	Normal	1,351	169 (12.50)	1,182 (87.50)	73.196	< 0.001
	Possible depression	167	43 (25.75)	124 (74.25)		
	Depression	212	72 (33.96)	140 (66.04)		
Sleep duration	< 8 h	1,043	165 (15.82)	878 (84.18)	1.504	0.471
	8–10 h	652	111 (17.02)	541 (82.98)		
	> 10 h	35	8 (22.86)	27 (77.14)		

x, mean; s, standard deviation; χ^2^, Chi-square; BMI, body mass index.

### 3.2 Multifactorial logistic regression analysis of sleep duration and depressive symptoms on school bullying

Multifactorial logistic regression analysis showed that adolescents with both possible depression (OR_1_ = 2.001, *p* = 0.002; OR_2_ = 5.176, *p* < 0.001) and depression (OR_1_ = 2.795, *p* < 0.001; OR_2_ = 6.520, *p* < 0.001) have increased the risk of exposure to one type and two types of school bullying compared to those normal group. Depression also increased the risk of exposure to three types of bullying in adolescents (OR = 15.312, *p* < 0.001). In addition, adolescents who sleep less than 8 h (OR = 0.118, *p* = 0.011) and between 8 and 10 h (OR = 0.166, *p* = 0.041) were less likely to exposure to three types of school bullying than those who sleep more than 10 h (Table 2).

**Table 2:** Multifactorial logistic regression analysis of sleep duration and depressive symptoms on school bullying.

School bullying	Comparison group	Reference group	OR	SE	Wald *χ^2^*	*p*	OR 95%CI	
							Lower	Upper
One type of bullying	Sleep duration <8 h	Sleep duration >10 h	1.170	0.620	0.064	0.800	0.347	3.947
	Sleep duration 8–0 h		1.533	0.624	0.469	0.493	0.452	5.203
	Possible depression	Normal	2.001	0.229	9.160	0.002	1.277	3.135
	Depression		2.795	0.197	27.236	< 0.001	1.900	4.113
Two types of bullying	Sleep duration <8 h	Sleep duration >10 h	0.313	0.654	3.152	0.076	0.087	1.128
	Sleep duration 8–10 h		0.351	0.674	2.406	0.121	0.094	1.317
	Possible depression	Normal	5.176	0.361	20.794	< 0.001	2.553	10.494
	Depression		6.520	0.329	32.489	< 0.001	3.422	12.422
Three types of bullying	Sleep duration <8 h	Sleep duration >10 h	0.118	0.840	6.467	0.011	0.023	0.613
	Sleep duration 8–10 h		0.166	0.877	4.186	0.041	0.030	0.927
	Possible depression	Normal	3.198	0.829	1.968	0.161	0.630	16.226
	Depression		15.312	0.535	26.026	< 0.001	5.367	43.682

OR, odd ratio; SE, standard error; χ^2^, Chi-square; 95%CI, 95% Confidence Interval.

### 3.3 Mediated model test for exposure to school bullying, sleep duration, and depression

**Figure 1:**
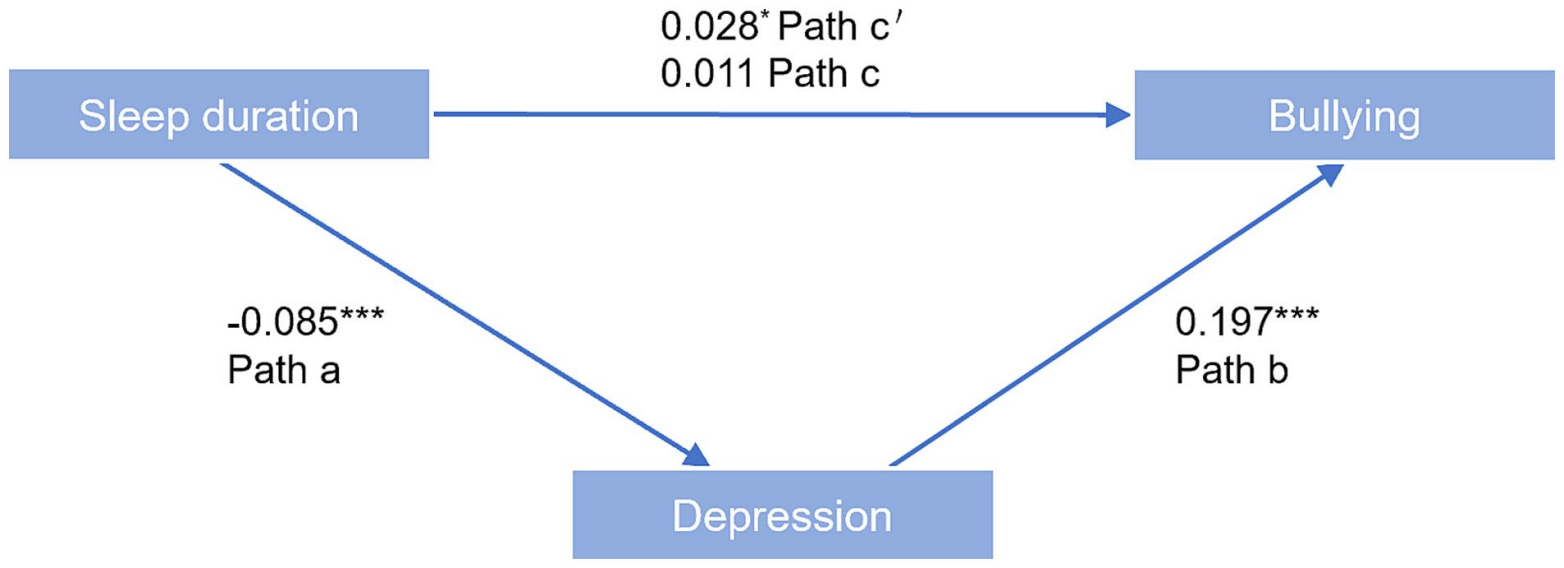
Mediated model of depressive symptoms on sleep duration and exposure to school bullying. Notes: Estimated path coefficients are referred to a, b, c, and c’. Values shown are standardized coefficients *p < 0.05, ***p < 0.001; total effect (c): the effect of sleep duration on exposure to bullying; indirect effect (ab): a represents the effect of sleep duration on depression; b represents the effect of depression on exposure to bullying after controlling for the effect of sleep duration; direct effect (c’): the direct effect of sleep duration on exposure to bullying.

Depressive symptoms were introduced as a mediating variable for mediating effect model based on the findings of regression analysis. The total effect (c) of sleep duration on exposure to school bullying was insignificant ( β = 0.019, *p* > 0.05). However, sleep duration significantly negatively predicted depression (β = −0.095, *p* < 0.001), while depression significantly positively predicted exposure to school bullying (β = 0.196, *p* < 0.001). However, the indirect effect (ab) was significant, with the absolute value of the direct effect (c’) exceeding that of the total effect (Figure 1; Table 3). The indirect and direct effects exhibited opposite signs. Depressive symptoms acted as a masking mediator between sleep duration and exposure to school bullying in adolescents. In Model 1, which did not adjust for any control variables (indirect effect = −0.019, 95% CI: −0.026 to −0.012), the indirect effect accounted for 50.00% of the direct effect (|ab/c’| = 0.500). Model 2 adjusted for control variables such as gender, age, ethnicity, BMI, and exercise intensity (indirect effect = −0.017, 95% CI: −0.026 to −0.009). The indirect effects accounted for 60.71% of the direct effects (|ab/c’| = 0.607) (Table 4).

**Table 3:** Mediating model tests for depressive symptoms in the relationship between sleep duration and exposure to school bullying.

Models	Dependent variables	Independent variables	R^2^	F	β	t
1	Exposure to school bullying	Sleep duration	0.002	2.614***	0.019	1.617
	Depressive symptoms	Sleep duration	0.023	40.903***	−0.095	−6.396***
	Exposure to school bullying	Depressive symptoms			0.196	10.475***
		Sleep duration	0.061	56.250***	0.038	3.240**
2	Exposure to school bullying	Sleep duration	0.012	3.435***	0.011	0.800
	Depressive symptoms	Sleep duration	0.024	7.036***	−0.085	−4.772***
	Exposure to school bullying	Depressive symptoms			0.197	10.574***
		Sleep duration	0.072	19.107***	0.028	2.028*

*p < 0.05, **p < 0.01, ***p < 0.001. Model 1 did not adjust for any control variables; Model 2 adjusted for control variables (gender, age, ethnicity, BMI, exercise intensity).

**Table 4:** Bootstrap test for sleep duration’s total, direct, and indirect effects on exposure to school bullying.

Models	Effect	β	SE	95%CI	
				Lower	Upper
1	Total effect	0.019	0.012	−0.004	0.042
	Direct effect	0.038	0.012	0.015	0.061
	Indirect effect	−0.019	0.004	−0.026	−0.012
2	Total effect	0.011	0.014	−0.017	0.039
	Direct effect	0.028	0.014	0.001	0.055
	Indirect effect	−0.017	0.004	−0.026	−0.009

SE, standard error; CI, confidence interval. Model 1 did not adjust for any control variables; Model 2 adjusted for control variables (gender, age, ethnicity, BMI, exercise intensity).

## 4 Discussion

Our findings revealed that depressive symptoms may serve as a risk factor for exposure to school bullying, whereas moderate and inadequate sleep duration appeared to act as protective factors against simultaneous exposure to all three types of school bullying. Notably, the impact of sleep duration on adolescents’ exposure to school bullying was influenced by the masking effect of depressive symptoms. Promoting appropriate sleep duration and fostering a positive, healthy mindset could therefore play a crucial role in reducing the prevalence of school bullying.

The findings revealed that 16.42% of adolescents experienced bullying in the past 30 days, indicating a concerning prevalence of school bullying in Xinjiang province, China and underscoring the urgent need for strengthened prevention and intervention measures. Additionally, we found that boys were significantly more likely to exposure to school bullying than girls, aligning with the findings of Morales et al. (2019) and Chen et al. (2022). There is a decline rate of exposure to school bullying with increasing grade levels. Older age emerged as a protective factor against bullying victimization, consistent with the findings of Tucker et al. (2014). As adolescents mature, the development of greater interpersonal skills may reduce their vulnerability to bullying at school, as improving social integration and social skills in adolescents may help to prevent the exposure to bullying (Sterzing et al., 2012). A study of adolescents in Ontario, Canada, using a BMI ≥ 25 kg /m^2^ as the criterion for obesity showed that obesity increased the odds of being bullied by 2 to 3 times (Kukaswadia et al., 2011). In our study, obesity (19.40%) was reported at a higher rate of exposure to school bullying than the normal (15.84%) and light (15.92%) groups of adolescents. The literature showed that during adolescence, obese body types were more likely to suffer rejection, social isolation, and malicious rumors in groups (Kilicaslan et al., 2023; Lian et al., 2018).

Multifactorial logistic regression analysis revealed that adolescents with possible depression had an increased the risk of exposure to one type and two types of school bullying, and depression all increased the risk of exposure to one to three types of school bullying. It may suggest that more severe depressive symptoms may increase the risk of exposure to more types of school bullying. A previous study found that impulsivity and obsessive-compulsive symptoms were also positively associated with many types of harassment (Liu et al., 2022). More severe depressive symptoms may increase social vulnerability, affecting a person’s ability or motivation to extricate themselves from risky situations such as exposure to bullying (Bhavsar et al., 2020). However, in contrast to the findings of most studies (Sampasa-Kanyinga et al., 2018; Yang et al., 2024; Na and Park, 2018), the results of our analyses suggested that insufficient and moderate sleep duration may be a protective factor for exposure to three types of school bullying among adolescents. A study of 67,821 Chinese students showed that meeting appropriate recommended sleep durations may be a protective factor against being bullied or bullying others (Chen et al., 2023). In addition, it may also not exclude bias due to numerical limitations due to small sample sizes in the long sleep duration group (> 10 h) for the current study, further expansion of the sample size is needed to validate the findings.

Further, we found that sleep duration was a significant positive predictor of exposure to school bullying, and a previous study revealed that excessive or unreasonable sleep duration can lead to obesity in adolescents (Xi et al., 2021). Hence, adolescents should need proper sleep duration, rather than a longer one being better. Additionally, sleep duration had a significant negative predictive effect on depressive symptoms, meaning that appropriate sleep duration was a protective factor for depressive symptoms. A recent study has confirmed the relationship between sleep duration and depression (Hysing et al., 2021). Depressive symptoms are a positive predictor of school bullying, so reducing depressive symptoms is beneficial in increasing adolescent wellbeing and reducing the likelihood of becoming victims of bullying. Overall, the mediating effects model constructed in the current study identified a possible masking effect of depressive symptoms in adolescents’ sleep duration and exposure to school bullying, suggesting two potential pathways for the association between sleep duration and exposure to school bullying. To account for the two-sided nature of the effect of sleep duration on exposure to school bullying, we hypothesized that sleepiness may impair the emotional regulation necessary to control aggression (O'Brien et al., 2011).

There are several limitations that should be considered in this study. Firstly, we were unable to reveal causal associations between sleep duration, depressive symptoms, and exposure to school bullying, and further prospective studies are needed to validate our findings. Secondly, this study design may not have investigated more possibly important confounding variables, such as social factors like cultural data, socioeconomic level, and family environment. However, we did our best to adjust for potential confounders in our analyses to minimize bias. Thirdly, like other mediated studies (Sampasa-Kanyinga et al., 2018), this study only captured the variable of sleep duration, so other vital aspects of sleep, such as sleep quality, structure, and problems. The introduction of potential bias from self-reported sleep duration also needs to be considered, and more objective assessments are needed in the future. Fourthly, our study sample is only from Xinjiang Province, China, which may limit the generalizability of our findings. Despite these limitations, our study contributes to understanding the influences on exposure to school bullying and provides helpful rationale and guidance.

## 5 Conclusion

In conclusion, our findings support that depressive symptoms may be a risk factor for exposure to school bullying. Depressive symptoms not only predicted sleep duration and exposure to school bullying but also moderated the relationship between sleep duration and bullying exposure. Excessive and insufficient sleep duration may both increase the risk of bullying through different pathways and mechanisms. These findings highlight two key directions for bullying prevention and intervention. First, addressing depressive symptoms is crucial: schools should monitor students for signs of depression, while primary care providers and educators should enhance mental health education, promote effective communication, and encourage physical activity. Second, interventions targeting the pathways in the masking model may also be effective. Parents can foster a harmonious family environment, help regulate adolescents’ sleep duration, and promote positive emotional experiences to reduce bullying incidents.

## Data Availability

The raw data supporting the conclusions of this article will be made available by the authors, without undue reservation.
